# Mannose Phosphate Isomerase Regulates Fibroblast Growth Factor Receptor Family Signaling and Glioma Radiosensitivity

**DOI:** 10.1371/journal.pone.0110345

**Published:** 2014-10-14

**Authors:** Aurélie Cazet, Jonathan Charest, Daniel C. Bennett, Cecilia Lopez Sambrooks, Joseph N. Contessa

**Affiliations:** Department of Therapeutic Radiology, Yale School of Medicine, New Haven, Connecticut, United States of America; Columbia University, United States of America

## Abstract

Asparagine-linked glycosylation is an endoplasmic reticulum co- and post- translational modification that enables the transit and function of receptor tyrosine kinase (RTK) glycoproteins. To gain insight into the regulatory role of glycosylation enzymes on RTK function, we investigated shRNA and siRNA knockdown of mannose phosphate isomerase (MPI), an enzyme required for mature glycan precursor biosynthesis. Loss of MPI activity reduced phosphorylation of FGFR family receptors in U-251 and SKMG-3 malignant glioma cell lines and also resulted in significant decreases in FRS2, Akt, and MAPK signaling. However, MPI knockdown did not affect ligand-induced activation or signaling of EGFR or MET RTKs, suggesting that FGFRs are more susceptible to MPI inhibition. The reductions in FGFR signaling were not caused by loss of FGF ligands or receptors, but instead were caused by interference with receptor dimerization. Investigations into the cellular consequences of MPI knockdown showed that cellular programs driven by FGFR signaling, and integral to the clinical progression of malignant glioma, were impaired. In addition to a blockade of cellular migration, MPI knockdown also significantly reduced glioma cell clonogenic survival following ionizing radiation. Therefore our results suggest that targeted inhibition of enzymes required for cell surface receptor glycosylation can be manipulated to produce discrete and limited consequences for critical client glycoproteins expressed by tumor cells. Furthermore, this work identifies MPI as a potential enzymatic target for disrupting cell surface receptor-dependent survival signaling and as a novel approach for therapeutic radiosensitization.

## Introduction

Receptor tyrosine kinases (RTKs) are key regulators of several critical biological processes including cell proliferation, migration, cell survival, and cell-cycle control [1]–[5]. This superfamily of transmembrane receptors share a similar molecular architecture, composed by an extracellular ligand-binding domain, a single membrane-spanning domain, and a conserved cytoplasmic tyrosine kinase domain [6]. RTKs are typically activated via ligand-induced dimerization, which stimulates kinase activation, and *trans*-phosphorylation of the carboxy-terminal tail, creating docking sites for phosphotyrosine-binding effectors that initiate intracellular signaling cascades. Because of their broad roles in many crucial cellular processes, RTK activation is normally under tight control, and loss of regulatory mechanisms thus lead to the genesis and progression of a variety of human cancers [2], [3], [7].

Fibroblast growth factor receptors (FGFR 1, 2, 3 and 4) compose a distinct subfamily of RTKs and are somewhat atypical in that they are stimulated by a combination of both ligand and heparan sulfate glycosaminoglycan co-factors [8]. Perturbation of the FGF/FGFR signaling axis through gene amplification, altered FGFR splicing, receptor overexpression, autocrine/paracrine loops, or gain-of-function mutations leads to enhanced tumorigenesis in a wide range of aggressive human cancers [4], [9]–[13]. FGFRs are a major focus for the development of new therapeutic approaches in patients whose tumors harbor enhanced FGF/FGFR signaling [4], [7], [9], and thus understanding the unique mechanisms that regulate this receptor family will be important for translating these targeted approaches to the clinic.

In addition to the genetic, transcriptional, and post-transcriptional mechanisms for RTK dysregulation, the contributions of protein N-linked glycosylation (NLG) to spatial distribution and clustering of receptors has also been identified as a novel mechanism for controlling RTK signaling [14]. NLG is a prominent endoplasmic reticulum (ER) co- and post-translational protein modification that plays an important role in the assembly and organization of receptors at the plasma membrane of multicellular organisms [15]. NLG can also affect receptor folding, stability, solubility, and maturation [15], and is thus essential for maintaining RTK function in tumor cells. Modification of core glycan structures, such as enhanced sialylation and fucosylation of the EGFR, suppresses dimerization and attenuates invasion of lung cancer cells [16]. Thus glycosylation is another key biosynthetic process that regulates RTK activation and cell signaling.

NLG is a sequence specific protein modification initiated on the cytoplasmic face of the ER membrane through transfer of GlcNAc-P from UDP-GlcNAc to dolichol phosphate (Dol-P) [17]. The resulting product, dolichol pyrophosphate *N*-acetylglucosamine (Dol-P-P-GlcNAc), is then used as the foundation for sequential addition of a GlcNAc, nine mannose (Man), and three glucose (Glc) carbohydrates which ultimately form a mature 14-carbohydrate lipid-linked oligosaccharide (LLO) [17], [18]. The predominant building block for LLO synthesis, mannose, is generated in the cell through conversion of fructose-6-phosphate to mannose-1-phosphate by two enzymes: mannose phosphate isomerase (MPI) and phosphomannomutase 2 (PMM2) [19]. Optimal function of these enzymes is therefore required for the completion of LLO synthesis in the ER and “en bloc” transfer by the oligosaccharyltransferase to NXS/T consensus protein sequences. These glycans can then be remodeled by glycosidases and glycosyltransferases in both the ER and Golgi apparatus [20].

We have previously demonstrated that disruption of NLG in glioma cells with tunicamycin, an inhibitor of dolichyl-phosphate N-acetylglucosamine-phospho-transferase (DPAGT1), down-regulates protein levels of multiple co-expressed RTKs [21]. NLG inhibition blocks RTK-dependent downstream survival signaling and also sensitizes tumor cells to ionizing radiation. However, the *in vivo* toxicity of tunicamycin prevents translation of this therapeutic strategy to the clinic [22]. We therefore explored additional gene therapeutic targets in the NLG biosynthetic pathway with the goal of disrupting RTK signaling and enhancing cell kill by radiation therapy. In this work, we report on the function of MPI, a regulator of mannose biosynthesis. We show that inhibition of MPI activity with either shRNA or siRNA in U-251 and SKMG-3 glioma cells specifically reduces dimerization and signaling of FGFR family members. Loss of FGFR signaling after MPI knockdown also revealed a migration defective phenotype as well as sensitivity to ionizing radiation *in vitro*, suggesting MPI is a potential therapeutic target for malignant glioma.

## Materials and Methods

### Antibodies and reagents

Mouse monoclonal antibody anti-phosphotyrosine (P-Tyr-100), anti-β-actin (8H10D10), anti-Akt, anti-phosphorylated Akt Ser473 (D9E), anti-phosphorylated mitogen-activated protein kinase antibody, anti-phosphorylated-p44/42 MAPK (Erk1/2), anti-phosphorylated EGFR (Tyr1173), anti-Met, anti-phosphorylated Met (Tyr1234/1235), anti-phosphorylated FGFR (Tyr653/654), anti-phosphorylated HERB3/ErbB3 (Tyr1289), anti-phosphorylated-FRS2-α (Tyr196), were purchased from Cell Signaling Technology, Inc. (Danvers, MA, USA). Anti-phosphotyrosine clone 4G10 was purchased from Millipore (Billerica, MA, USA). Anti-MPI was from Sigma-Aldrich (St. Louis, MO, USA). Anti-human FGFR2 antibody (α isoform) was purchased from R&D Systems (Minneapolis, MN, USA), and the anti-human FGFR2 monoclonal antibody (M01) clone 1G3 was purchased from Abnova (Walnut, CA, USA). Anti-ErbB3 (G-4), anti-FGFR1 (C-15) and anti-FRS2 (H-91) antibodies were purchased from Santa Cruz Biotechnology, Inc. (Santa Cruz, CA, USA). Anti-EGFR antibody was kindly provided by Joseph Schlessinger (Yale University, New Haven, CT, USA). Anti-rabbit and anti-mouse IgG conjugated to horseradish peroxidase were purchased from Millipore and GE Healthcare (Chalfont St. Giles, Buckinghamshire, UK), respectively. Alexa Fluor 543-conjugated goat anti-rabbit IgG and Alexa Fluor 488-conjugated goat anti-mouse IgG were purchased from Molecular Probes/Life Technologies (Grand Island, NY, USA).

### Cell lines

Cell culture reagents were purchased from Gibco (Grand Island, NY, USA). The human glioblastoma cell lines U-251 MG and SKMG-3 were gifts from Ted Lawrence (University of Michigan, Ann Arbor, MI, USA) and have previously been described [57]. All cells were routinely grown in RPMI-1640 medium supplemented with 10% fetal bovine serum (FBS) and 100 units/mL penicillin-streptomycin in monolayer cultures maintained at 37°C in an atmosphere of 5% CO2. Stable MPI or scrambled shRNA-expressing cell lines were generated through lentiviral transduction with mission short hairpin RNA lentiviral plasmids (Sigma-Aldrich). Following HEK293T cell co-transfection of packaging plasmids and shRNA plasmids, supernatants were collected and used to infect the U-251 parental cell line. Cultures with shRNA plasmid expression were selected with puromycin (2 µg/mL) and clones were isolated by serial dilution. Stable shRNA-expressing clones were maintained in culture with 2 µg/mL puromycin.

### RNA interference experiments

U-251 MG or SKMG-3 glioblastoma cells were seeded in 6-well plates and grown to 40–50% confluency in serum-containing medium. Cells were then transfected with 50, 100 or 200 pmol of the MPI targeting siRNA (Integrated DNA Technologies, Wallingford, CT, USA; (MPI (stealth) 5′-GCAUCUGUCAGAGCAAGAGACCAGG-3′)) or a non-silencing siRNA sequence (Universal Negative Control, Integrated DNA Technologies) using Lipofectamine 2000 (Invitrogen, Carlsbad, CA, USA) following the manufacturer's instructions. 48 hours later, cells were collected for immunoblotting experiments in order to analyze the knockdown efficiency. Two cycles of siRNA were performed to achieve maximal knockdown. Cells were transfected twice with corresponding siRNAs at t = 0 h and t = 48 h and were collected 24 h later for cell growth, migration, and clonogenic assays, and 48 h later for Western blot experiments.

### Western blot and Immunoprecipitation

For cell signaling experiments, cells were starved overnight in serum-free RPMI medium prior to stimulation with either 50 ng/mL human recombinant FGF-1, 30 ng/mL human recombinant HGF/SF (both from PeproTech, Rocky Hill, NJ, USA), or 10 ng/mL EGF (Sigma-Aldrich). Heparin was used at a final concentration of 50 ng/mL. The FGFR inhibitor PD173074 (EMD4Biosciences, Philadelphia, PA, USA) was used at a final concentration of 1 µM.

For media transfer experiments, supernatants from U-251 control cells grown under identical conditions were added to U-251 scramble or MPI shRNA clones for 30 min at 37°C. Cells were harvested by scraping in cold phosphate-buffered saline (PBS) and subjected to centrifugation (10.000 g, 10 min). The pellets were then resuspended in lysis buffer (25 mM Tris-HCl pH 7.4, 10 mM EDTA, 15% glycerol, 0.1% Triton X-100, protease inhibitor tablet (Roche Diagnostics; Indianapolis, IN, USA) and phosphatase inhibitor cocktails 2 and 3 (Sigma-Aldrich)). The supernatants were assessed for protein concentration using the Bio-Rad RC protein assay kit II (Bio-Rad, Hercules, CA, USA), and equal amounts of protein were subjected to SDS-PAGE, transfer to PDVF membranes (Millipore), and western blot analysis.

For immunoprecipitation experiments, 200 µg of lysates were immunoprecipitated with 2 µg of the FGFR2α-specific antibody (clone MAB6841) or an anti-ErbB3 (clone G-4) with agitation at 4°C for 2 hours. Antibody bound proteins were recovered after adding 30 µL of Protein A/G PLUS- Agarose Immunoprecipitation Reagent (Santa-Cruz Biotechnology) for 1 h at 4°C. Beads were centrifuged and washed with the following buffers: lysis buffer, lysis buffer supplemented with 500 mM NaCl, lysis/buffer/TNE (10 mM Tris/HCl, 150 mM NaCl, and 1 mM EDTA, pH 7.4) and finally TNE alone. Western blot analyses were performed using a global phospho-tyrosine antibody (4G10) or the anti-phospho-ErbB3 (clone 21D3), respectively. The Western blot membranes were then stripped and re-probed for total FGFR2α (MAB6841) or ErbB3 (G-4).

For phospho-RTK array analysis, cells were cultured for 48 hours in 6-well plates in serum-containing medium, starved in serum-free medium overnight, and then lysed in NP-40 lysis buffer (1% NP-40, 20 mM Tris-HCl (pH 8.0), 137 mM NaCl, 10% glycerol, 2 mM EDTA, protease and phosphatase inhibitor cocktail tablets (Roche)). The human Phospho-RTK array kit (R&D Systems) was used according to the manufacturer's protocol. Briefly, the arrays were incubated overnight at 4°C with 200 µg of total protein extract, washed three times, followed by detection with a horseradish-peroxidase-conjugated phospho-tyrosine antibody.

### FGFR2 Cross-linking and Immunoprecipitation

Cell cultures were plated in 100 mm plates and serum starved in serum-free medium overnight. After stimulation with FGF-1 (50 ng/mL) for 30 min, cells were scraped, centrifuged, and washed three times with ice-cold PBS. The chemical cross-linker BS^3^ (Pierce Biotechnology; Rockford, IL, USA) was added to a final concentration of 4 mM and the reaction was quenched by adding 20 mM Tris-HCl, pH 7.4, for 15 min. Cross-linked cells were lysed and immunoprecipitated with an anti-FGFR2α-specific antibody (clone MAB6841). The precipitates were subjected to 7.5% SDS-PAGE, transferred to PVDF, and analyzed as described above.

### Immunofluorescence

For immunofluorescence, U-251 shRNA cell lines were grown on glass coverslips to 60% confluence. Cell cultures were washed with PBS and fixed with 4% (w/v) formaldehyde in PBS for 30 min at 37°C. After washing with PBS, cells were permeabilized with 0.2% v/v Triton X-100 in PBS for 10 min, rinsed there times in PBS and then treated with 5% w/v bovine serum albumin for 1 h to block nonspecific binding sites. Cells were then incubated overnight at 4°C with primary antibodies and for 1 h at room temperature with the appropriate secondary antibodies. All antibodies were diluted in PBS containing 5% w/v bovine serum albumin. Rabbit anti-EGFR pAb (1∶2,000) and mouse anti-FGFR2 mAb (1∶200) were used as primary antibodies. Alexa Fluor 543-conjugated goat anti-rabbit IgG (1∶1,000), Alexa Fluor 488-conjugated goat anti-mouse IgG (1∶1,000) we used as secondary antibodies. Confocal cellular images were captured with an inverted Zeiss LSM 510 Pascal laser confocal microscope (Carl Zeiss, Jenna, Germany), using a 63/1.4 Plan-Apochromat objective.

### MPI enzyme assay

The activity of MPI was determined by modification of previously published methods [23] using MPI as the rate-limiting step for conversion of Mannose-6-Phosphate to 6-phospho-gluconate. This is accomplished through supplementation of excess phospho-glucose isomerase (PGI) and glucose-6-phosphate dehydrogenase (G6PDH) in an *in vitro* reaction with measurement of NADP to NADPH conversion through absorbance changes at 340 nM. Cells were scraped and sonicated in 25 mM Tris buffer with protease and phosphatase inhibitors prior to removal of cell debris by centrifugation and determination of protein concentration. 15 µg of total protein was then incubated with PGI, G6PDH, and 0.7 mM MgCl2, NADP, and Man-6-P. Reactions were performed at room temperature and monitored for 60 minutes.

### Proliferation assays

Growth rates were determined by CellTiter 96 NonRadioactive Cell Proliferation Assay (Promega; Madison, WI, USA) according to the manufacturer's directions. U-251 control and MPI-shRNA clones (2×10^3^) were seeded in 96-wells plates (Corning Incorporated, Corning, NY, USA) and grown in culture medium containing 10% or 0.1% FBS. Cell numbers were estimated after 0, 1, 2, 3, 4 and 5 days by adding MTT [(3-(4,5-Dimethylthiazol-2-yl)-2,5-diphenyltetrazolium bromide] reagent to the wells 4 hours before taking the spectrophotometric reading (absorbance at 570 nm). For RNA interference assays, 2×10^3^ SKMG-3 cells were seeded in 96-well plates in RPMI-1640 serum-free medium 1 day after the transfection with siRNA sequences, then cultured 3 days before the assessment of cell growth.

### In vitro scratch wound-healing assays

Wound healing assays were used to evaluate cell motility of MPI-siRNA glioma cells. U-251 control, U-251 MPI-shRNA clones, SKMG-3 scrambled and SKMG-3 MPI-siRNA used for *in vitro* scratch wound-healing assays were seeded in 6-well plates and cultured until they reached confluence. After 24 hours of starvation in serum-free RPMI medium, a scratch wound was introduced with a 10 µL pipette tip, and the media was then replaced with fresh medium containing 1% FBS supplemented with the indicated inhibitors, growth factor, or with DMSO as control. After 18 hours, cells at the same positions along the scratch wound were photographed using an inverted phase-contrast microscope. Images are representative of the results obtained in at least four independent experiments.

### Clonogenic survival

For U-251 shRNA cell lines, clonogenic survival was determined by standard colony formation assays [21]. For siRNA experiments, transfection with MPI or scramble sequences were performed as described previously followed by irradiation with 0, 2, and 4 Gy 24 hours following transfection. Survival curves were generated using the linear quadratic equation.

### Data analysis

All values are expressed as the mean ± standard error of the mean (SEM). Statistics following a Student t distribution were generated using the t-test. P values of <0.05 were considered as statistically significant. NIH Image J software (http://rsb.info.nih.gov/ij/) was used to analyze and quantify scratch wound assays according to established methods [24]. At least 100 distance readings for each sample were performed. Dose enhancement ratios were calculated at a surviving fraction of 37% and represent the ratio of dose required from control cells *vs.* cells with MPI knockdown.

## Results

### MPI inhibition alters RTK activity in human glioma cells

**Figure 1:**
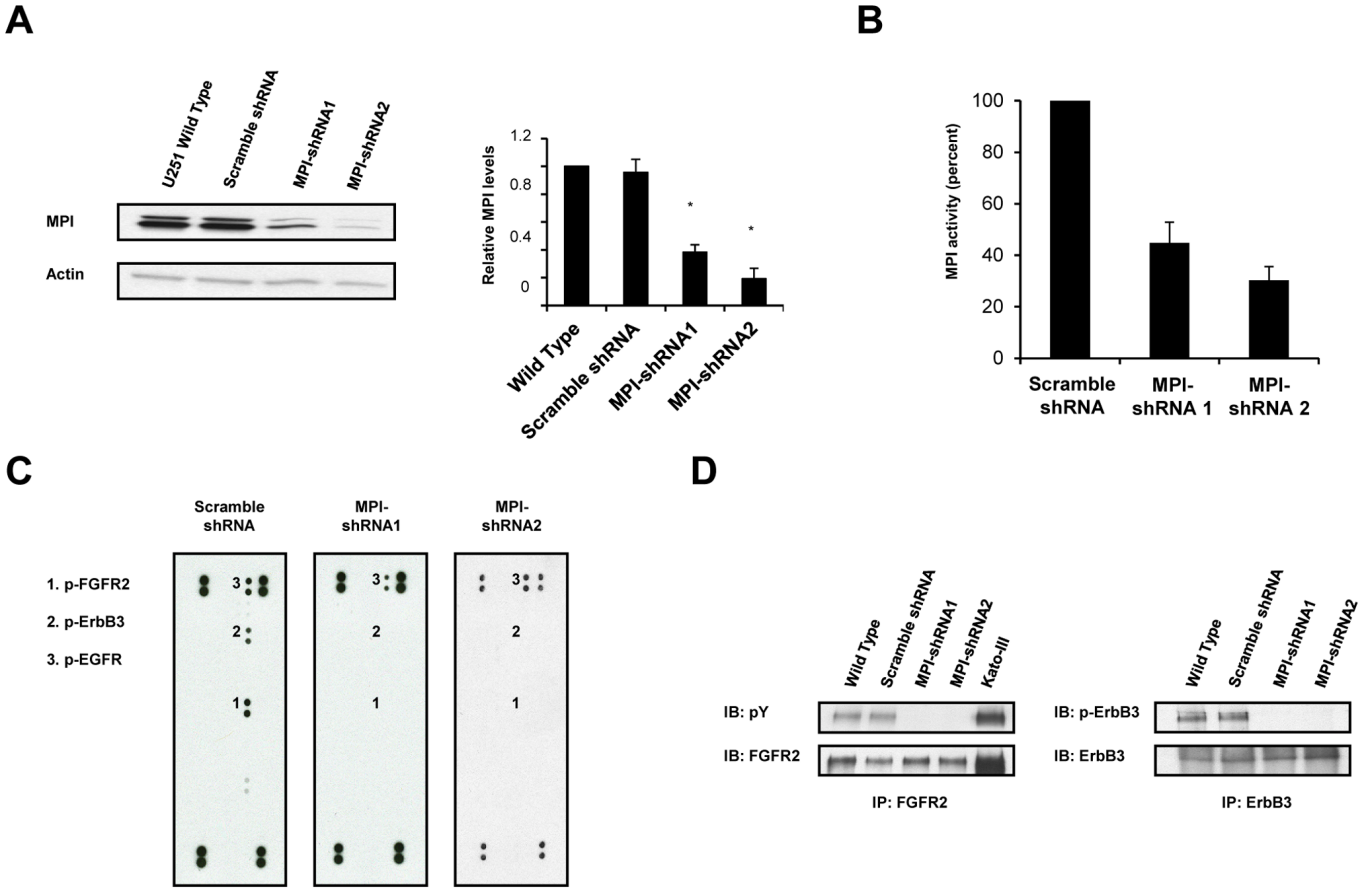
MPI knockdown reduces RTK phosphorylation in human glioblastoma cells. Stable U-251 MG cell lines with scramble shRNA or MPI-shRNA were generated with lentiviral infection as described in Materials and Methods. **A.** Western blots demonstrating decreased MPI protein expression with actin used as a loading control. Relative MPI levels were quantified using Image J software (right panel). Asterisks (*) indicate a significant decrease of MPI expression. (For all densitometric quantifications n = 3; ± s.e.m; Student's t-test *p≤0.05. **B.** *In vitro* biochemical analysis and quantification of MPI enzymatic activity in MPI-shRNA knockdown or control cell lines. **C.** RTK phospho-array results for U-251 control or MPI-shRNA knockdown cell lines. Membranes are spotted with individual RTKs in duplicate and detected following incubation with a phospho-tyrosine specific antibody. Receptors of interest (1) FGFR2, (2) ErbB3, and (3) EGFR are indicated on the left. Duplicate corner spots are phospho-tyrosine positive controls. **D.** Immunoprecipitation (IP) and Western immunoblot (IB) analysis of FGFR2 and ErbB3 phosphorylation from U-251 control and MPI-shRNA clones. The KatoIII gastric carcinoma cell line was used as a positive control for FGFR2 [56]. Blots were stripped and re-probed to insure equal loading of FGFR2 or ErbB3.

To determine the functional importance of MPI expression for RTK signaling in gliomas, we used lentiviral transduction to generate U-251 cells expressing either MPI-shRNA knockdown or a scramble shRNA sequence (Fig. 1). Two clones with approximately 70% decrease in MPI activity and a corresponding decrease in MPI protein expression were isolated and used for further experimentation (Fig. 1A and 1B). RTK phosphorylation status was then examined using phospho-RTK arrays to compare control and MPI knockdown cell lines. In this screening assay, MPI knockdown altered the RTK activation profile of U-251 cells (Fig. 1C). Control cells displayed a strong and constitutive phosphorylation of the FGFR2 receptor, but tyrosine phosphorylation of this receptor was absent in MPI-shRNA knockdown cell lines. Furthermore, a decrease in tyrosine phosphorylation of ErbB3, an RTK with an inactive TK domain, was also observed in the MPI-shRNA clones. To validate the phospho-RTK array results, FGFR2 and ErbB3 receptors were specifically immunoprecipitated (IP) from U-251 control and MPI-shRNA lysates followed by detection of tyrosine phosphorylation through western blot analysis. As shown in Figure 1D, this method confirmed that MPI knockdown abolished the constitutive FGFR2 and ErbB3 phosphorylation observed in both wild type and control U-251 cells. While the results show that MPI function is required for activation of FGFR2 and ErbB3, they also indicate that EGFR phosphorylation was not affected by MPI knockdown, and suggest that the functionality of FGFR is sensitive to low levels of MPI activity.

### MPI knockdown suppresses FGFR2 dimerization

**Figure 2:**
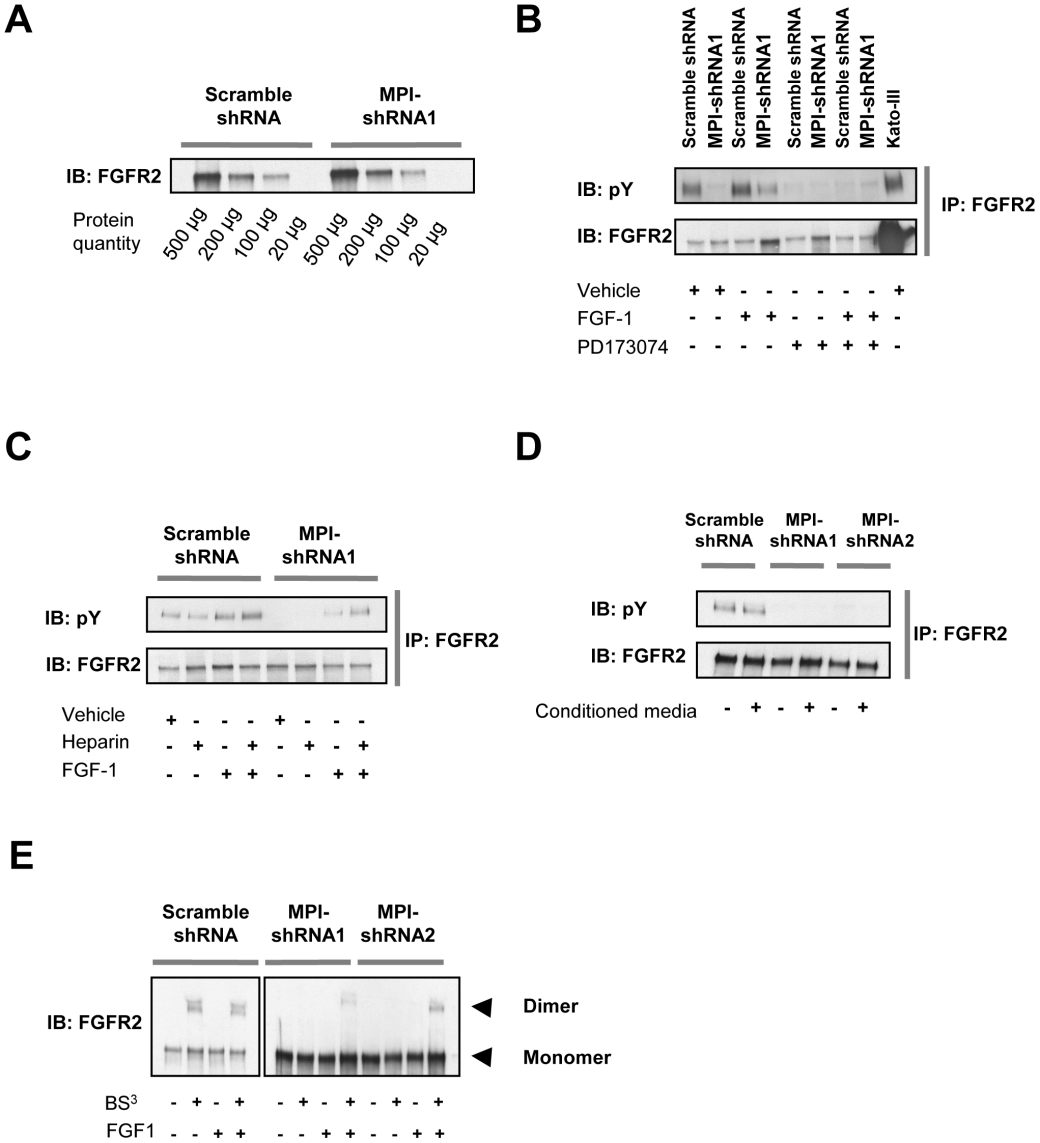
MPI knockdown inhibits FGFR2 dimerization. **A.** FGFR2 protein level analysis. FGFR2 was immunoprecipitated from decreasing amounts of protein lysate to compare relative abundance of the receptor between the cell lines. **B.** FGF-1 activation of FGFR2 in control and MPI knockdown U-251 cell lines. Cells were incubated with combinations of FGF-1 ligand (50 ng/mL), and/or 1 µM PD173074 and FGFR tyrosine kinase inhibitor. FGFR2 immunoprecipitations were then analyzed with western blot for tyrosine phosphorylation. The Kato-III cell line was used as a positive control for FGFR2 expression. **C.** Effect of heparin on FGF-1 induced FGFR2 phosphorylation. Control or MPI-shRNA knockdown U-251 cells were pretreated with or without 50 ng/mL of heparin for 1 hour and then stimulated with or without 50 ng/mL of FGF-1 for 30 min. FGFR2 immunoprecipitates were then analyzed for tyrosine phosphorylation by western blot. **D.** FGFR2 phosphorylation following media transfer. U-251 cell lines were incubated with conditioned media from U-251 control cells for 30 min at 37°C to evaluate for a secreted activator of the receptor, and FGFR2 immunoprecipitates were analyzed. **E.** FGFR2 dimerization analysis. U-251 control and MPI-shRNA knockdown clones were stimulated with or without FGF-1 (50 ng/mL) for 30 min and then incubated with the non-cell permeable bis-sulfosuccinimidyl cross linker substrate (BS^3^) as described in Materials and Methods. Total protein adjusted lysates were immunoprecipitated and analyzed by SDS-PAGE for FGFR2 dimerization. Arrowheads indicate FGFR2 monomer or dimer formation. Data for each panel is representative of three independent experiments.

We have previously demonstrated that inhibition of DPAGT1, the first step in lipid-linked oligosaccharide synthesis, reduces RTK phosphorylation through a reduction of newly synthesized receptors [21]. We were therefore surprised to find no difference of FGFR2 receptor levels between control and MPI knockdown cell lines and confirmed this observation with FGFR2 IP experiments. Figure 2A shows that IP experiments using a constant amount of antibody with decreasing protein concentrations did not demonstrate appreciable differences in terms of FGFR2 expression between the cell lines.

We next tested whether FGF1, a potent FGFR2 ligand [25], was able to stimulate the receptor in the setting of MPI knockdown. FGFR2 was weakly stimulated by FGF1 (and completely blocked by the FGFR specific tyrosine kinase inhibitor PD173074) suggesting its presence at the plasma membrane (Fig. 2B). Because the FGFR family of receptors is regulated by heparan sulfate (HS), which serves as a co-factor to promote receptor activation, we also examined whether treatment with heparin (a structural analog of HS; [26]–[28]) could increase FGFR2 stimulation by FGF1 in MPI knockdown cells. We found that heparin alone was not sufficient to activate FGFR2 but that combined stimulation with FGF1 and heparin did augment FGFR tyrosine phosphorylation similar to control cells (Fig. 2C). This finding raised the possibility that decreased FGFR2 phosphorylation was caused by the loss of a secreted ligand, however, media transfer experiments did not restore FGFR2 phosphorylation arguing against this hypothesis (Fig. 2D).

**Figure 3:**
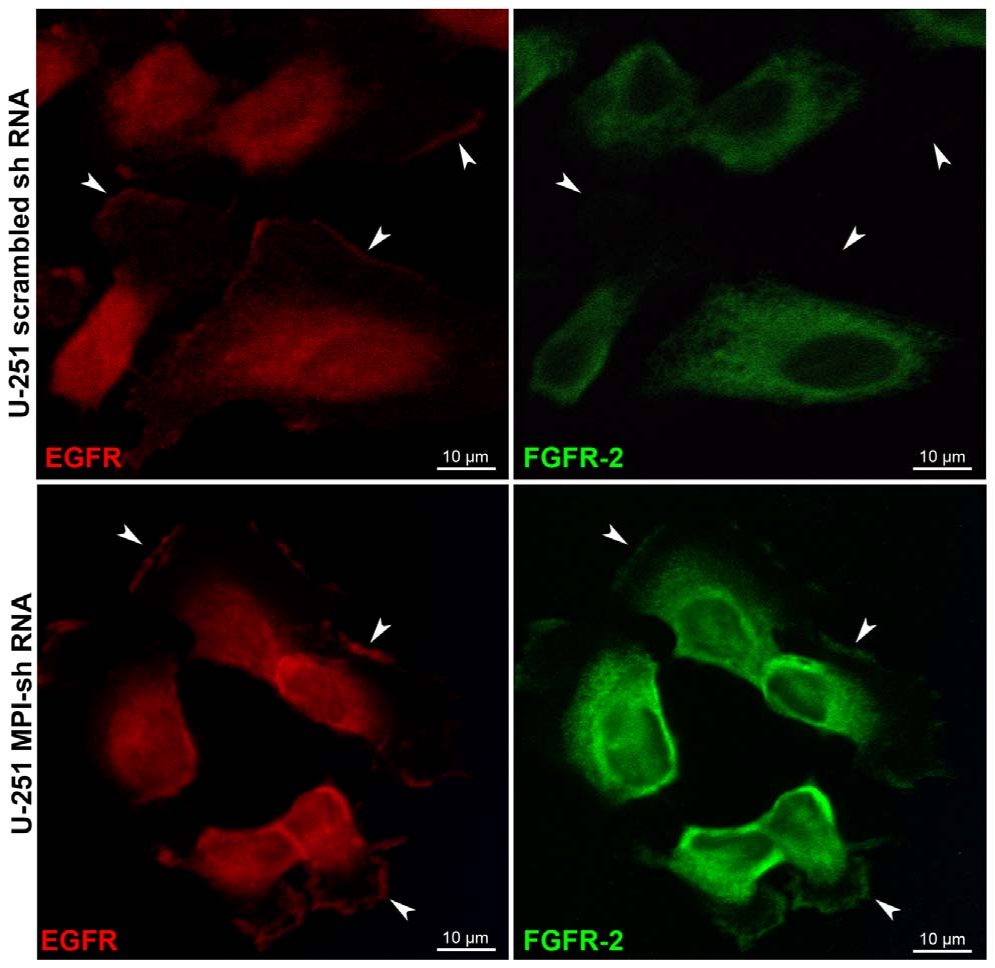
MPI knockdown restored FGFR2 membrane localization. The localization of EGFR and FGFR2 was analyzed by confocal immunofluorescence microscopy in U-251 scramble and MPI shRNA cell lines. EGFR (red) and FGFR2 (green) membrane localization was determined for each sample.

To more clearly understand the consequences of MPI knockdown on FGFR2 function, we evaluated receptor dimerization in U-251 cells through BS^3^ cross-linking experiments. In control cells, FGFR2 dimerization was observed both with and without FGF1 stimulation, and like phosphorylation, appeared to be constitutive. In comparison FGFR2 dimerization was absent in the non-stimulated state in MPI knockdown cells lines and was weakly restored by FGF1 stimulation (Fig. 2E). We then performed confocal microscopy to evaluate for potential differences in RTK localization. The findings, consistent with constitutive dimerization and phosphorylation, showed that FGFR2 is not located at the plasma membrane in control U-251 cells (Fig. 3, upper right). In U-251 cells with MPI knockdown, however, FGFR2 membrane localization was restored (Fig 3, lower right). These findings are in contrast to the EGFR which displayed membrane localization in both control and MPI knockdown cell lines (Fig 3, left). Together these experiments demonstrate that knockdown of MPI results in impaired FGFR2 dimerization, loss of phosphorylation, and redistribution of the inactive receptor to the plasma membrane.

### MPI inhibition reduces FGFR family signaling

**Figure 4:**
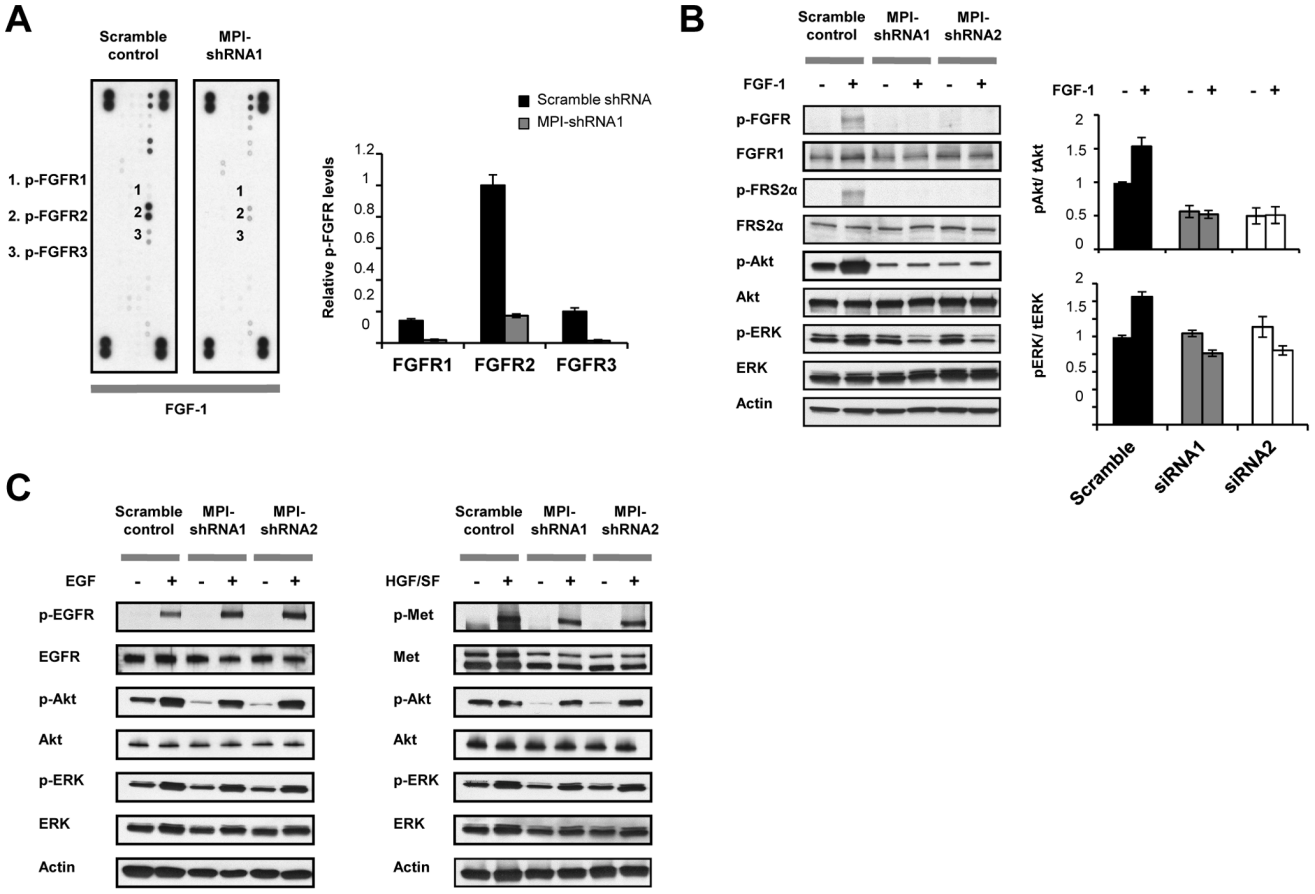
MPI knockdown reduces FGFR family RTK activation and signaling. **A.** RTK phospho-array analysis of FGF-1 (50 ng/mL) stimulated U-251 control or MPI- shRNA knockdown cell lines was performed as described previously. Tyrosine phosphorylated (1) FGFR1 (2) FGFR2 and (3) FGFR3 are indicated on the left. Signal intensity was analyzed using Image J software to compare relative levels of FGFR phosphorylation between the cell lines (right panel). **B.** MPI knockdown blocks FGF-1 induced FGFR signaling. Western blot analysis of control and MPI-shRNA knockdown cell lines stimulated with 50 ng/mL FGF-1 was performed to determine induction of FGFR1, FRS2, Akt, and ERK phosphorylation. Actin expression was used as a control for protein loading. Quantification of Akt and ERK phosphorylation relative to total protein was determined as in 3A and are representative of three experiments (right panel). **C.** MPI knockdown does not block EGF or HGF/SF RTK signaling. Western blot analysis of control and MPI-shRNA knockdown cell lines stimulated with 10 ng/mL EGF or 30 ng/mL HGF/SF were performed to determine induction of EGFR, Met, Akt, and ERK phosphorylation.

FGFR2 is a member of the FGFR RTK family composed of FGFR1, FGFR2, FGFR3, and FGFR4. We next investigated whether MPI knockdown also blocked activation of other co-expressed FGFR family members. Using the phospho-RTK array, U-251 RTK activation profiles were analyzed by densitometry following FGF1 stimulation. We found that 50 ng/mL FGF1 weakly activated FGFR1 and FGFR3 in control U-251 cells but was not sufficient to induce tyrosine phosphorylation of these FGFRs in U-251 cells with MPI knockdown (Fig. 4A). These results suggest that the effects of MPI knockdown are not limited to FGFR2 alone.

To validate these findings, we next performed western blot analyses on FGFR1, FRS2, Akt and MAPK in the control and MPI knockdown cell lines to further investigate the effects of MPI knockdown on FGFR signaling. Using a specific anti-phospho-FGFR antibody, we found that FGFR1 tyrosine phosphorylation was not activated by FGF1 in MPI knockdown cell lines (Fig. 4B). Furthermore the loss of FGFR activation also correlated with a failure to transduce downstream signaling to FRS2 or MAPK. MPI knockdown also reduced Akt phosphorylation under both non-stimulated and FGF-stimulated experimental conditions in comparison to the scramble control, suggesting that FGFR family signaling is a key regulator of Akt in the U-251 cell line.

To further characterize the selectivity of MPI knockdown on specific RTKs, we investigated the effects of MPI knockdown on EGFR and MET, two other RTKs co-expressed by the U-251 cell line. We found that neither EGF stimulation of EGFR/MAPK/AKT phosphorylation nor HGF stimulation of Met/MAPK/AKT phosphorylation was blocked by MPI knockdown (Fig. 4C). Together these results demonstrate that MPI knockdown appears to specifically affect FGFR family signaling but does not reduce signaling through all RTK families.

**Figure 5:**
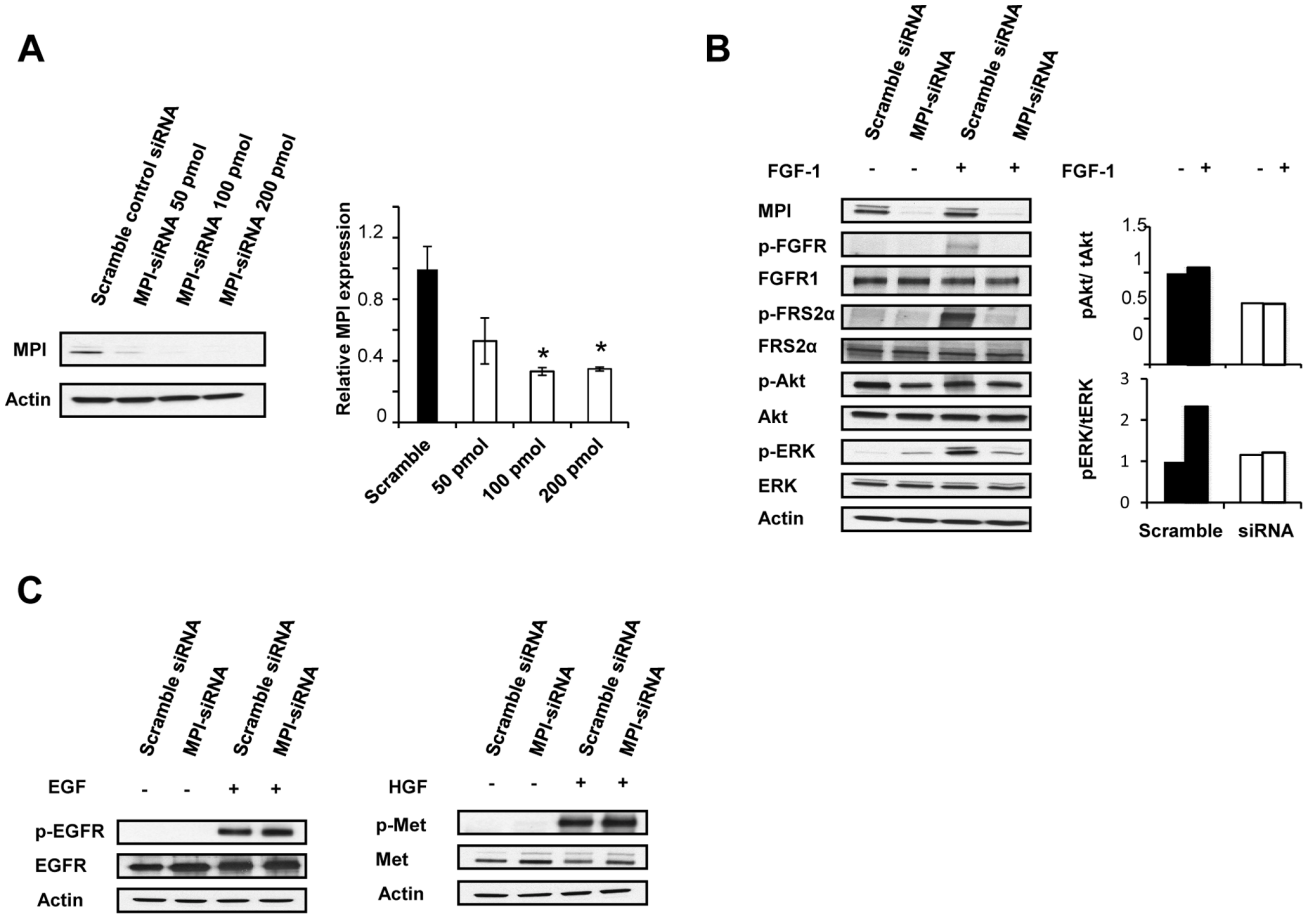
MPI knockdown blocks FGFR1 signaling in SKMG-3 human glioma cells. **A.** Efficiency of MPI-siRNA knockdown. SKMG-3 cells were transfected with increasing concentrations of MPI targeted siRNA or with a control, non-silencing siRNA sequence. MPI expression was then evaluated by western blot and data for three experiments was quantified as above. 100 pMol siRNA was used for all further experiments. Asterisks (*) indicate a significant decrease of MPI expression after siRNA experiment compared to the expression in the control cells. *p≤0.05. **B.** MPI siRNA inhibits FGFR1 phosphorylation and signaling. Western blot analysis of MPI-siRNA knockdown in SKMG-3 cells stimulated with 50 ng/mL FGF-1 was performed to determine induction of FGFR1, FRS2, Akt, and ERK phosphorylation. Actin expression was used as a control for protein loading. Quantification of Akt and ERK phosphorylation relative to total protein was determined for two experiments as described previously (right panel). **C.** MPI siRNA does not inhibit EGFR or Met activation. Western blot analysis of control or MPI-siRNA knockdown following stimulation with 10 ng/mL EGF or 30 ng/mL HGF/SF was performed to determine induction of EGFR or Met phosphorylation.

In order to generalize the role of MPI in modulating FGFR activation in glioma, MPI expression was targeted in SKMG-3 cells through transfection with a specific siRNA sequence designed to target MPI [19]. Transient siRNA transfection demonstrated near complete reduction of MPI expression in comparison with scramble control siRNA by western blot at 100 pmol (Fig. 5A), and this concentration was used for all further siRNA experiments. Because the SKMG-3 cell line expresses FGFR1, we analyzed receptor phosphorylation and signaling after siRNA transfection both with and without FGF1 stimulation. Our results show that transient MPI knockdown blocks FGF1-induced tyrosine phosphorylation of FGFR1 as well as downstream activation of FRS2 and MAPK (Fig. 5B). In SKMG-3 cells FGF1 does not activate Akt, and correspondingly, MPI knockdown also did not reduce Akt phosphorylation levels. The effects of MPI knockdown were also evaluated for ligand-induced activation of EGFR and Met, and consistent with the findings in U-251 cells, MPI knockdown did not diminish activation of either RTK (Fig. 5C). The results of siRNA knockdown of MPI in SKMG-3 cells therefore provide further experimental evidence for the role of MPI in regulating FGF family receptor signaling in glioma.

### MPI regulates glioma cell migration

**Figure 6:**
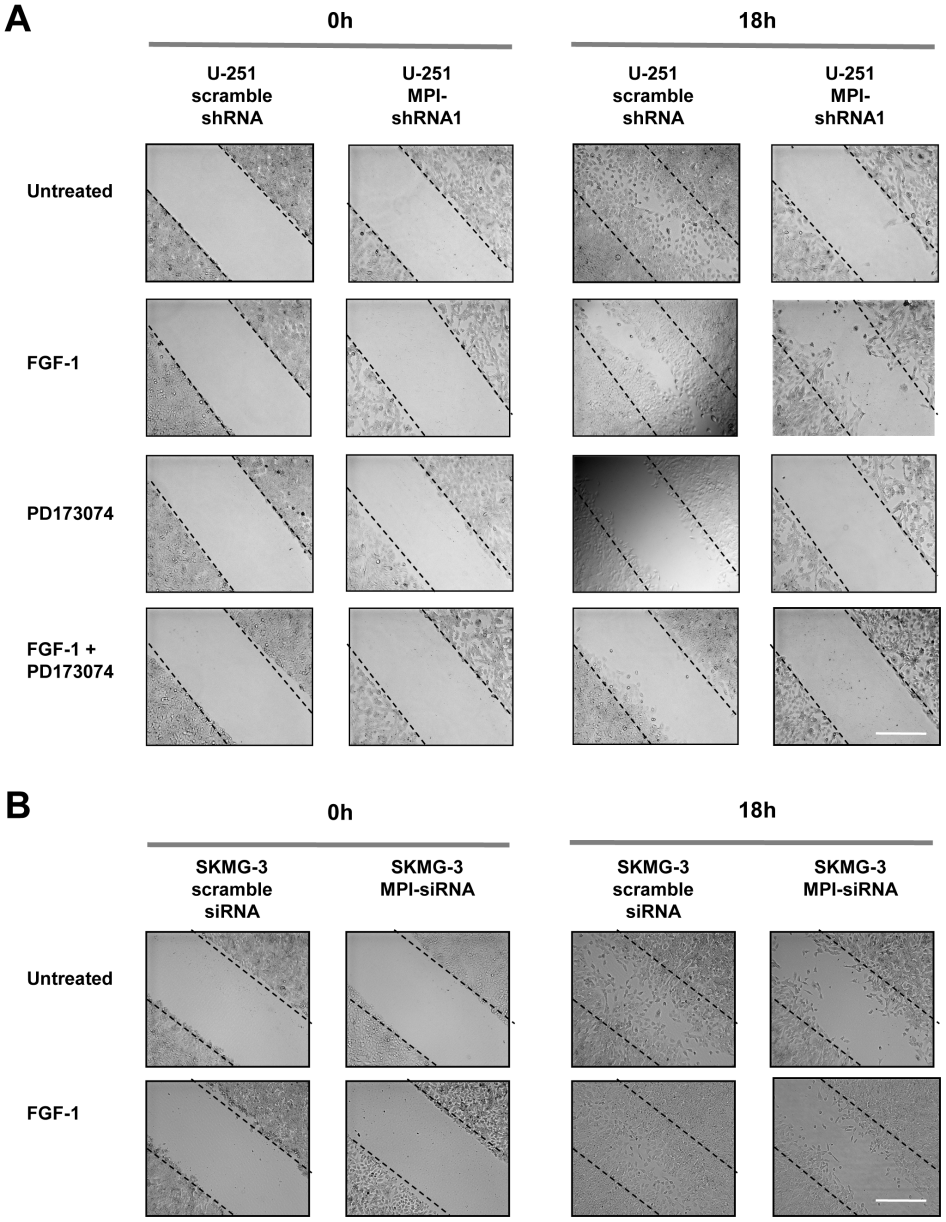
MPI knockdown decreases migration of human glioblastoma cells. **A.** Scratch-wound assay for shRNA knockdown in U-251 cells. A cell-free area was introduced to confluent cultures with a pipette tip; cells were then incubated with combinations of FGF-1 ligand (50 ng/mL), and/or 1 µM PD173074 and pictures were taken at t = 0 and t = 18 hours after the scratch wounding. Images are representative of four independent experiments. Scale bar: 400 µM. **B.** Wound healing assays in SKMG-3 cells. Images are representative of three experiments following transfection with MPI specific or non-specific siRNA sequences and incubation with 50 ng/mL of FGF-1 as described in the Material and Methods section. Scale bar: 400 µM.

FGF receptors are known to regulate cellular migration, and we therefore investigated the effects of reduced MPI activity on glioma cell migration using *in vitro* scratch wound-healing assays. Under these *in vitro* culture conditions, control U-251 cells migrated into the wound by 18 hrs. However a striking difference was found for U-251 MPI shRNA clones. These cells did not migrate into the wound after 18 hours of culture (Fig. 6A). U-251 cell migration was partially rescued with FGF1 stimulation (38% of controls), consistent with western blots showing partial FGFR activation, and was blocked with PD173074. Together these experiments demonstrate loss of U-251 migratory ability through manipulation of FGFR signaling (Fig. 6A). The result of MPI siRNA knockdown on cell migration was also investigated in the SKMG-3 glioma cell line, and like the U-251 cells, we found impaired migration after MPI knockdown with partial rescue by FGF1 (Fig. 6B). These results suggest that one consequence of reduced MPI activity is inhibition of FGFR-dependent cell migration.

### MPI regulates glioma cell proliferation and radiosensitivity

**Figure 7:**
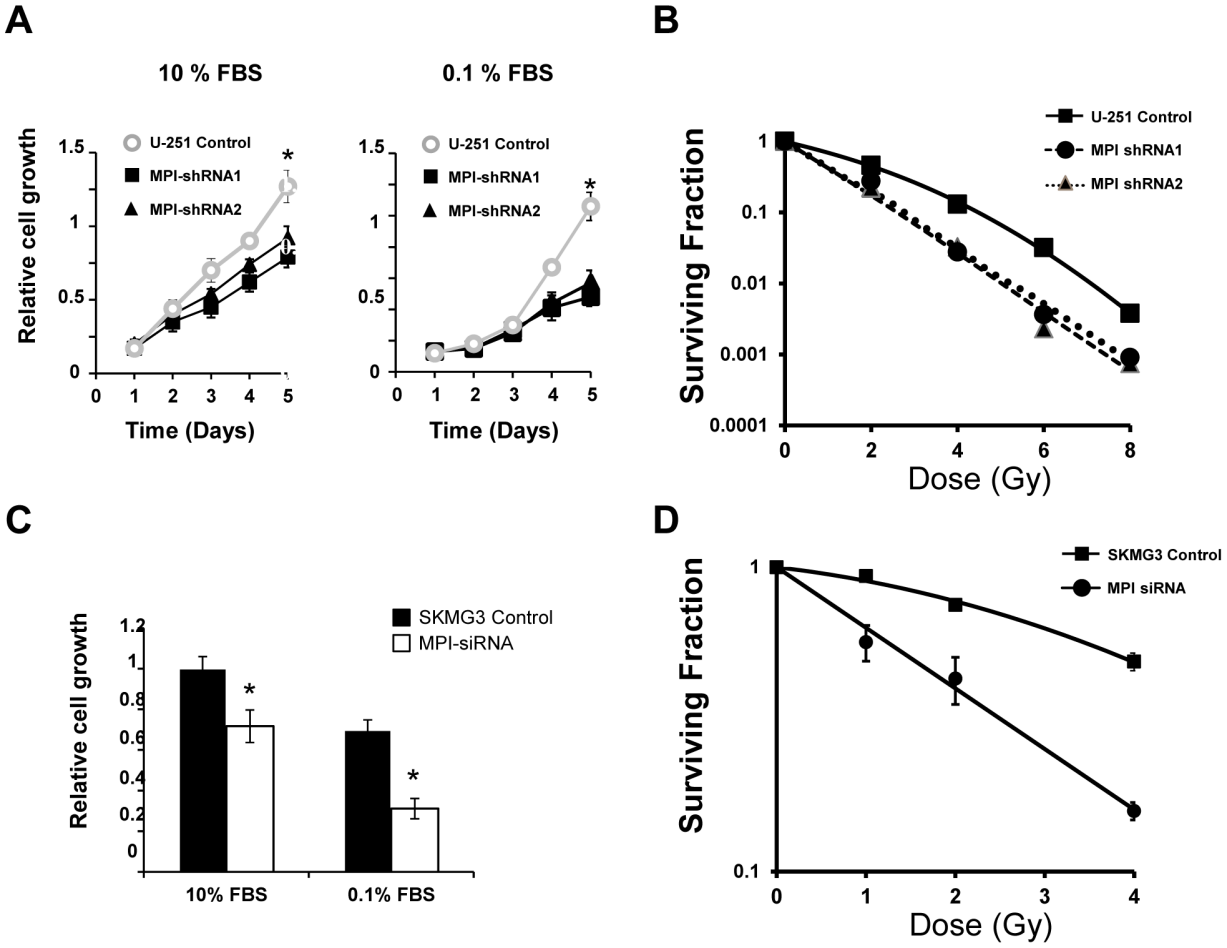
Effect of the MPI knockdown on glioma proliferation and radiosensitization. **A.** U-251 control and MPI-shRNA clones were cultured in RPMI medium containing 10% or 0.1% FBS for 5 days. Cell growth was determined by MTT assay. Data is representative of three independent experiments. *p≤0.05, MPI-shRNA knockdown U-251 cells *vs* scramble control cells. **B.** Clonogenic survival analysis for U-251 scramble and MPI knockdown cell lines. Data represents the average of three experiments performed in triplicate. **C.** Proliferation assay after MPI-siRNA experiment in SKMG-3 cells. One day after transfection, SKMG-3 cells were seeded in 96-well plates and cell growth was then analyzed after 48 h as described in Material and Methods. Absorbance values were normalized to the control cells transfected with a scramble sequence and cultured in 10% or 0.1% FBS containing medium. *p≤0.05, MPI-siRNA knockdown SKMG-3 cells *vs* scramble control cells. **D.** Clonogenic survival analysis for SKMG-3 cells transfected with control or MPI siRNA. Data represents the average of 2 experiments performed in triplicate.

We also investigated the effects of MPI inhibition on cell proliferation using MTS assays in both U-251 and SKMG-3 cell line models. MPI knockdown slowed proliferation of U-251 cells by 18-30% (30±7% and 18±3% at 3 days for clones 1 and 2 respectively) and reduced SKMG-3 cell proliferation by 30±8% at 2 days (Fig. 7A and 7C). Similar results were obtained by direct cell counting experiments (data not shown). The growth effect was more pronounced with low serum conditions (0.1%) suggesting that glioma cell proliferation is more sensitive to loss of MPI function in the setting of reduced exposure to growth factors.

Finally, we directly evaluated radiation responses of glioma cells in the setting of decreased MPI activity through clonogenic survival analysis of U-251 and SKMG-3 cells (Fig. 7B and 7D, respectively). In U-251 cells, shRNA knockdown of MPI produced significant reductions in cell survival for each dose tested (p<0.05, with the exception of the 2 Gy dose in one MPI knockdown clone p = 0.058). The DERs were 2.1±0.1 for each clone. Similar results were found in SKMG-3 glioma cells after MPI knockdown with siRNA. A significant radiosensitization was observed at each dose tested (p<0.05) and the DER was greater than 2.0.

## Discussion

To investigate the contributions of glycosylation to RTK function, we generated a model system with shRNA knockdown of MPI. This gene is required for the synthesis of mannose-6-phosphate, a phospho-sugar precursor used for synthesis of GDP-mannose and mannosylphosphoryl dolichol (Dol-P-Man), both carbohydrate precursors predominantly utilized by the cell for protein glycosylation. The successful establishment of a MPI deficient cell line thus provides a novel tool for glycobiology studies as few mammalian cell lines with NLG gene defects exist and MPI deficient models are otherwise limited to fibroblasts derived from patients with congenital disorders of glycosylation type IB (CDG1B) [19], [20], [29]–[33] These immortalized fibroblasts grow relatively slowly in culture but have no explicit phenotype with respect to the expression or function of specific proteins [19], [31]–[33]. In contrast, our MPI knockdown model using a glioma cell line revealed a specific dysfunction for a family of glycosylated proteins (FGFRs), and is to our knowledge the first demonstration of a cell signaling deficit associated with an NLG gene defect.

Using both shRNA and siRNA techniques in U-251 and SKMG-3 glioma cell lines, we examined the extent to which MPI knockdown affected RTK signaling. RTK phospho-arrays were employed to screen activity of co-expressed receptors and our subsequent experiments demonstrated a reduction in FGFR1 and FGFR2 phosphorylation in glioma cell lines. The effect of MPI knockdown appears to be relatively specific for the FGFR family of RTKs as there was no observed decrease in either baseline or ligand-stimulated activation of both the EGFR and MET RTKs. The reduction of FGFR activation by MPI knockdown also resulted in decreased downstream signaling through FRS2 and either AKT or MAPK in the U-251 and SKMG-3 glioma cell lines, respectively. Furthermore, like the FGFR small molecule inhibitor PD170374 [34], MPI knockdown both abrogated glioma cell motility and migration in a scratch-wound assay and reduced cellular proliferation. These findings therefore link MPI function to the regulation of cellular processes that contribute to glioma progression and aggressiveness. Previously we have proposed that inhibition of protein glycosylation provides a novel strategy for disrupting RTK signaling and enhancing radiation-induced cell death. In agreement with this hypothesis, we now show that MPI deficiency interferes with FGFR activation and glioma radiation responses, and identifies MPI as a potential NLG enzymatic target for cancer therapy.

The impairment of FGFR dimerization after MPI knockdown provides a mechanistic understanding for the observed reduction in FGFR activation. While RTKs are classically considered to be ligand-induced signaling receptors, ligand independent RTK dimerization and signaling is well known to occur during carcinogenesis [13]. This phenomenon is best exemplified by ErbB2, an orphan receptor that drives cellular programs for cell growth, proliferation, invasion, and survival in epithelial malignancies [35]–[38]. Through crosslinking experiments we demonstrated that MPI knockdown reduced constitutive dimerization of FGFR2, and analysis of FGFR2 phosphorylation showed a commensurate reduction in phosphorylation. Confocal microscopy experiments with U251 cells also demonstrated that constitutively dimerized FGFR2 predominately localized to an intracellular compartment and that disruption of dimerization and phosphorylation by MPI knockdown resulted in redistribution of FGFR2 to the plasma membrane. However, because FGFR2 can still be stimulated by FGF1 plus heparin in the setting of MPI knockdown, we conclude that MPI knockdown does not completely eliminate function of the receptor, but impairs formation of stable FGFR dimers.

Disruption of mannose precursor biosynthesis could regulate FGFR dimerization through both direct and indirect paths. Altered glycosylation through changes in site occupancy [39], [40] or glycotype [16], [41], [42] have been demonstrated to modify dimerization or activation of the FGFR, EGFR, and PDGFR [42]. In our experiments, no change in FGFR molecular weight was observed following MPI knockdown, suggesting that any of these direct changes in FGFR glycosylation would be limited to individual glycosylation sites. Loss of single glycosylation sites in RTKs can lead to decreased amounts of protein or even receptor activation [39], [58], and therefore this mechanism is less likely to explain our findings. Instead we speculate that indirect modulation of FGFR dimerization through either co-factors such as heparan sulfate proteoglycans [43], [44], facilitators of dimerization such as the galectins [45], or interactions with FGFR phosphatases may underlie the observed changes in FGFR dimerization, signaling, and cellular localization.

In previous work we demonstrated that inhibition of DPAGT1, an enzyme that catalyzes addition of the first carbohydrate to the elongating LLO, produced a pleiotropic effect on protein levels of multiple co-expressed RTKs through blockade of glycosylation [21], [22]. The limited effect of MPI knockdown on RTK activation profiles presented herein was therefore somewhat surprising and provides a new insight on the cellular consequence of modulating specific enzymes required for protein glycosylation. Current models of NLG propose that a defect in any of the 26 defined enzymatic steps in LLO biosynthesis or the OST [20] results in the absence of glycosylation. In opposition to this view, mammalian cell line models of ALG6 homolog deficiency (α-1,3 glucosyltranferase) and MPDU1 deficiency have demonstrated that immature LLOs (Man5GlcNAc2 and Man9GlcNAc2) can be transferred to substrate proteins at reduced rates [46], [47] and that NLG is therefore not a simple serial process where loss of one enzymatic step completely incapacitates the glycosylation machinery. Our findings using a glioma cell line add to our understanding of NLG deficiencies and suggest that significant reduction of an NLG enzymatic activity may impact specific and susceptible groups of glycosylated proteins such as RTKs. This data also provides compelling evidence for the further study of deficits in enzymes required for NLG precursor biosynthesis in order to define the spectrum of biochemical and cellular effects on both normal and malignant cell types.

The contributions of FGFR signaling to malignant glioma progression and survival have been well established [4], [9], [48]–[50]. The more recent discovery of FGFR-TACC translocations in glioblastoma has reinforced the concept that FGFR activation can drive signaling required for gliomagenesis [51]. FGF-induced activation of FGFR has also been identified to promote glioma cell migration and proliferation [50], [52], regulate the PTEN tumor suppressor [53], and enhance MAPK and AKT phosphorylation [49]. Our data is in agreement with these previous studies and supports the importance of FGFR signaling in glioma. In this work we demonstrate that MPI knockdown reduces FGFR activity, a finding that provides a molecular mechanism for MPI's effect on FGFR-dependent signaling, proliferation, and cell survival after radiation therapy.

Novel targets in oncology should exploit a therapeutic ratio between tumor cells and normal tissues. Targeting the function of an essential gene like MPI may therefore seem counter-intuitive. However, elevated mannose precursor biosynthesis may be more important for rapidly dividing cells or developing tissues, as suggested by a recent mouse genetic study using hypomorphic alleles of PMM2 (the enzymatic step following MPI for mannose-1-phosphate synthesis). In this study exogenous mannose rescue was used to demonstrate that although mannose-1-phosphate sugar donors are required for development, they may be required at only very low levels after development [54]. Thus, enzymes like MPI that are crucial for enabling cell surface receptor signaling, but with low activity requirements in adult tissues, may be ideal targets for developing cancer therapeutic approaches. Our data provides the groundwork for investigating this concept for MPI and other genes required for LLO elongation. Furthermore, with the recent interest in developing small molecule inhibitors of MPI [19], [55], our work gives the initial impetus for structural optimization and testing of these drug-like scaffolds as potential cancer therapies.

In summary, targeting NLG to disrupt cell surface receptor function and reduce survival signaling is a novel approach for therapeutic radiosensitization. Previous strategies were limited by toxicity and a non-selective effect on glycosylated proteins that traverse the ER [22]. We therefore investigated MPI, the enzyme that regulates mannose precursor biosynthesis, to further explore the feasibility of this strategy. We show that MPI knockdown disrupts the function of transmembrane glycoproteins, inclusive of but likely not limited to FGFR family RTKs, and this work suggests that a partial inhibitory effect on NLG could be a therapeutic strategy for the treatment of malignancy.
